# Elective egg freezing patients may benefit from increasing the maximal daily gonadotropin dose above 300IU

**DOI:** 10.1186/s12958-022-01049-3

**Published:** 2022-12-19

**Authors:** Raoul Orvieto, Adva Aizer, Bozhena Saar-Ryss, Lilach Marom-Haham, Meirav Noach-Hirsh, Jigal Haas, Ravit Nahum

**Affiliations:** 1 Department of Obstetrics and Gynecology, Chaim Sheba Medical Center, Ramat Gan, Israel; 2 Sackler Faculty of Medicine, Tel-Aviv University, Tel Aviv-Yafo, Israel; 3 The Tarnesby-Tarnowski Chair for Family Planning and Fertility Regulation, Sackler Faculty of Medicine, Tel-Aviv University, Tel Aviv-Yafo, Israel; 4 Department of Obstetrics and Gynecology, Barzilai Medical Center, Ashkelon, and Ben Gurion University School of Medicine, Beer Sheva, Israel

**Keywords:** IVF, Ovarian stimulation, Gonadotropin daily dose, Elective egg freezing, Mature oocytes

## Abstract

**Objective:**

Nowadays, patients attempting social/elective egg freezing has spread globally. Ovarian stimulation (OS) with high daily gonatotropin doses, are commonly offered to this group of patients, aiming to achieve the maximal oocytes cohort with minimum IVF cycle attempts. We aim to assess the IVF-ET outcome, and specifically the oocyte yield, of patients undergoing two successive IVF cycle attempts for elective egg freezing (EEF), and whether changing the daily gonadotropin dose in the second IVF cycle attempt, affect the outcome.

**Patients and methods:**

All women admitted to our IVF unit for social/EEF, who underwent 2 consecutive IVF cycle attempts, with only those who used in the first attempt a starting daily gonadotropin dose of 300 IU were included. Ovarian stimulation characteristics, duration of OS, number of retrieved oocytes, number of mature oocytes were assessed and compared between the 1st and the 2nd IVF cycle attempts, and between the different daily gonadotropin doses and the oocyte yields in the 2nd cycle attempt (increase, decrease or no change).

**Main outcome measures:**

Oocytes and mature oocytes yield in the 2^nd^ as compared to the 1^st^ IVF cycle attempt.

**Results:**

A reduced oocyte yield in the 2nd cycle attempt was observed in those who highly responded in the 1st attempt, regardless the daily dose in the 2nd cycle attempt (whether it was increased, no change and decreased). Moreover, the proportion of patients with same or more oocytes in the 2nd IVF cycle attempt was significantly lower in patients with high peak E2 levels, compared to those with peak E2 levels < 9175 pmol/L. Among patients with high peak E2 (> 9175 pmol/L), those who achieved a lower oocytes yield in the 2nd IVF cycle attempt had lower basal Day-3 FSH/LH ratio (1.5 + 0.5 vs 1.8 + 0.8, *p* < 0.03) and higher oocyte (range: 7–28, median:10; vs range: 2–15, median:7) and mature oocytes yields. With a cut-off of 9 oocytes, 78.8% of those with > 9 oocytes and 61.8% of those with < 9 oocytes will achieve lower/higher oocytes yield in the 2nd IVF cycle attempt, respectively.

**Conclusions:**

Ovarian stimulation with high daily gonatotropin doses (300 IU) should be offered to patients attempting social/EEF. Moreover, in their 2nd IVF cycle attempt, those with high peak E2 (> 9175 pmol/L) in the 1st attempt, and basal Day-3 FSH/LH ratio < 1.5 and/or more than 9 oocytes retrieved, should receive same OS protocol with no change in the daily gonadotropin dose.

## Introduction

Ovarian stimulation (OS) is considered a key factor in the success of in vitro fertilization-embryo transfer (IVF-ET), enabling the recruitment of multiple oocytes and, thereby, resulting in multiple embryos [1]. While several societies (e.g. WHO, NICE, RANZCOG) have previously discussed OS for IVF/ICSI, the most recent and updated guidelines was published at 2019 by the European Society for Human Reproduction and Embryology (ESHRE) special interest group (SIG) Reproductive Endocrinology [2]. The later aimed to provide clinicians with evidence-based information on the different options for OS for IVF/ICSI.

When the ESHRE SIG guideline on OS for IVF/ICSI discussed the role of daily gonadotropin dose, they quoted a Cochrane meta-analysis by Lensen, et al. [3], reporting on no significant difference in ongoing pregnancy rate or number of oocytes retrieved, while using 300 IU versus 400/450 IU daily dose of gonadotropins. The ESHRE SIG therefore concluded that since there is unlikely to be significant benefit with doses > 300 IU daily, a gonadotropin dose higher than 300 IU *is not recommended* for predicted poor responders.

When searching the literature regarding the maximal daily gonadotropin dose, there are differences in the reported threshold, which constitutes “high" or "maximal" daily dose. A prospective trial by Klinkert et al. [4] demonstrated that patients with poor response didn’t benefit from a higher starting dose of gonadotropins (150 IU vs. 300 IU) with the same number of oocytes retrieved in both groups and same pregnancy rate. Moreover, Manzi et al. [5] and Stadtmauer et al. [6] could not observe any advantage, by increasing the dosage of the gonadotropins, on the number of oocytes retrieved, or pregnancy rate. Similarly, Karande et al. [7] showed in their retrospective study that by increasing the dosages of gonadotropins from 300 to 450 IU/day, neither the number of oocytes retrieved nor pregnancy were increased.

On the other hand, Hofmann et al. [8] described better outcomes in women with poor ovarian response after raising the dosage of gonadotropins to 300 IU/day, with higher pregnancy and reduced cancellation rates. In another study of poor responder patients, Land et al. [9] demonstrated that the use of high daily dosage of gonadotropins (300 IU) resulted with significantly more follicles and number of oocytes retrieved compared to the previously cycle treated with 225 IU, but with comparable number of embryos and low similar pregnancy rates (3.2%). Moreover, our group evaluated the role of increasing the daily dose to 450 IU, in patients who received a daily dose of 300 IU during their previous OS for IVF [10]. While comparing IVF cycles using daily gonadotropin dose of 300 IU to 450 IU (*n* = 117), no in-between group differences were observed, except for significantly higher yield of oocytes retrieved. Moreover, cycles using daily gonadotropin dose of 450 IU resulted in 7.7% live-birth rate [10].

What about normal, or high responder patients? Ben-Rafael and colleagues [11] found a direct correlation between the administered daily dose of gonadotropin and follicle-stimulating hormone (FSH) levels reached in the blood, and that FSH levels started to rise from the 1st day of gonadotropin administration to a plateau after several days in normal responders, but continuing to rise in the high-responder group. The same group [12] also compared the hormonal profiles of ovulatory high and low responder patients who were treated with different daily doses (225 and 150 IU) of gonadotropins in successive IVF cycles. The magnitude of the ovarian response was found to be altered by changing the dose of hMG in high responders, but not in normal responders [12], suggesting an inability to alter the temporal individuality of endocrine profiles by varying the dose of gonadotropin. In this study [10], while the increases in hormone levels accompanying a high response to hMG could be dampened by lowering the dose, hormone concentrations were not influenced by changing the dose of hMG in low responders.

During the last decade, and particularly since 2018 following the American Society for Reproductive Medicine (ASRM) endorsement of social oocyte cryopreservation [13], elective egg freezing (EEF) has spread globally, with Israel among the first country to introduce EEF in IVF clinics. In Israel, EEF for non-medical reasons is allowed for women age 30 to 41yrs. Women might undergo up to four IVF cycle attempts or up to 20 mature oocytes cryopreserved- whichever is earlier. While, according to the Israel Health Law, almost every woman up to age 45 may legally seek *government-subsidized* infertility treatment with her own oocytes for as many cycles as necessary for the birth of two children, EEF for non-medical reason is not subsidized. Ovarian stimulation with high daily gonatotropin doses, are commonly offered to this group of patients, aiming to achieve the maximal oocytes cohort with minimum IVF cycle attempts.

Prompted by the aforementioned observations, we aim to assess the IVF-ET outcome, and specifically the oocyte yield, of patients undergoing two successive IVF cycle attempts for EEF, and whether changing the daily gonadotropin dose in the second IVF cycle attempt, affect the outcome.

## Patients and methods

We reviewed the computerized files of all consecutive women admitted to our IVF unit, between February 2018 and February 2022, and reached the ovum pick-up (OPU) stage. The elimination of bias in this selection, for the purposes of this study, was achieved by including only women undergoing OS, using in the first IVF cycle attempt a starting daily gonadotropin dose of 300 IU. The study was approved by our institutional review board (SMC-9589–22).

All women underwent the multiple dose GnRH-antagonist protocol with GnRH-agonist for triggering final follicular maturation. In all 1^st^ IVF cycle attempts, the starting daily gonadotropins dose was 300 IU, which was later adjusted according to serum E2 levels and vaginal ultrasound measurements of follicular diameter obtained every 2 or 3 days. In the 2^nd^ IVF cycle attempt, the gonadotropin daily dose was determined by the treating physician and largely dependent on the fashion at the time.

In order to evaluate whether changing the daily gonadotropins dose will improve IVF outcome, we conducted further sub-analyses, according to the change in the daily dose in the 2^nd^ cycle attempt (increase, decrease or no change), and according to women that their oocyte yield was increased, decreased, or no change.

Data on patient age and infertility-treatment-related variables were collected from the files. Ovarian stimulation characteristics, duration of OS, number of retrieved oocytes, number of mature oocytes were assessed and compared between the 1^st^ and the 2^nd^ IVF cycle attempts.

Results are presented as means ± standard deviations. Differences in variables were statistically analyzed by student’s paired t-test, Fisher’s exact test and chi-square test, as appropriate. A *p* value of less than 0.05 was considered significant.

## Results

Two hundred and seventeen consecutive women undergoing two successive IVF cycle attempts were evaluated. Women age and body mass index were 35.7 ± 2.2yrs and 23.3 ± 4.6 kg/m^2^, respectively. The IVF cycle characteristics are shown in Table 1. While there were no between- cycle differences in the duration of OS, peak E2 and progesterone levels or the number of oocytes retrieved, during the 2nd IVF cycle attempt women used a significantly higher daily gonadotropin dose (390 ± 101 vs 300 ± 0; *p* < 0.000) and yielded more follicles ≥ 13 mm in diameter on day of trigger (9.6 ± 5.3 vs 8.7 ± 4.2; *p* = 0.05). and more mature oocytes (8.96 ± 5.19 vs 8.04 ± 4.7; *p* = 0.05).

**Table 1:** IVF cycle characteristics

OS variables	1^st^ IVF attempt	2^nd^ IVF attempt	*p*-value
Daily Gonadotropin dose (IU)	300 ± 0	390 ± 101	< 0.001
Duratin of stimulation (days)	9.9 ± 1.6	10.2 ± 1.7	NS
Number of follicles > 13 mm in diameter	8.7 ± 4.2	9.6 ± 5.3	< 0.05
Peak E2 (pmol/L)	8839 ± 5554	8966 ± 6222	NS
Peak progesterone (nmol/L)	2.8 ± 1.7	2.8 ± 1.6	NS
Number of oocytes retrieved	10.6 ± 6.4	11.4 ± 7.0	NS
Number of mature oocytes retrieved	8.04 ± 4.7	8.96 ± 5.19	< 0.05

*OS* Ovarian stimulation, *NS* Not significant

### Women who achieved same or more, as compare to those with reduced oocyte yield in the 2nd IVF cycle attempt.

In the 2nd IVF cycle attempt, 96 (44.2%) women achieved a lower oocyte yield, 13 (6%) achieved the same and 108 (49.7%) an increased oocyte yield. While comparing the OS characteristics during the 1st IVF cycle attempt, those who achieved a lower oocyte yield in the 2nd attempt were significantly younger (35.2 ± 2.1yrs vs 36.1 ± 2.2yrs; *p* < 0.01), had significantly higher number of follicles > 13 mm in diameter (10.3 ± 4.1 vs 7.5 ± 3.8; *p* < 0.001) and higher peak E2 (10,369 ± 6377 pmol/L vs 7615 ± 4458 pmol/L; *p* < 0.001) levels on day of trigger, and significantly more oocytes (14.0 ± 6.8 vs 7.8 ± 4.6; *p* < 0.001) and mature oocytes (10.8 ± 4.8 vs 5.9 ± 3.2; *p* < 0.001) yields, as compared to women who achieved same or more oocytes in the 2nd attempt. No in between group differences were observed in the duration of OS, total dose of gonadotropin used, nor peak progesterone levels. While assessing the OS variables during the 2^nd^ IVF attempt, those who achieved a lower oocyte yield in the 2nd attempt received significantly higher daily dose of gonadotropins (415 ± 88 IU vs 369 + 106 IU, *p* < 0.001), had significantly lower number of follicles > 13 mm in diameter (6.7 ± 3.7 vs 11.9 ± 5.2; *p* < 0.001) and lower peak E2 (6842 ± 4581 pmol/L vs 10,665 ± 6829 pmol/L; *p* < 0.001) and progesterone (2.5 ± 1.1 nmol/L vs 3.0 ± 1.8 nmol/L; *p* < 0.03) levels on day of trigger, and significantly lower oocytes (7.2 ± 4.4 vs 14.8 ± 6.9; *p* < 0.001) and mature oocytes (5.7 ± 3.3 vs 11.5 ± 5.0; *p* < 0.001) yields, as compared to women who achieved same or more oocytes in the 2nd attempt.

In the 2^nd^ IVF cycle attempt, 23 (10.6%) women receive a lower daily gonadotropin dose, 60 (27.6%) received the same dose and 134 (61.7%) an increased daily dose. The oocyte yields in each subgroup of women (increased, no change and decreased daily dose) are shown in Table 2.

**Table 2:** The oocyte yields in the 2nd IVF cycle attempt, according to changes in the daily gonadotropin dose

	# of patients	Oocytes yield in the 2^nd^ attempt	
		Relative to the 1^st^ attempt	# of patients (%)
Lower dose	23	Less	1 (4%)
		Same	6 (26%)
		More	16 (69.5%)
Same dose	60	Less	16 (26.7%)
		Same	4 (6.6%)
		More	40 (66.7%)
Increased daily dose	134	Less	74 (55.2%)
		Same	8 (6%)
		More	52 (39%)

### Women with reduced daily gonadotropin dose in the 2^nd^ IVF cycle attempt

While comparing the OS characteristics during the 1^st^ IVF cycle attempt, those who achieved a lower oocyte yield in the 2^nd^ attempt had significantly higher number of follicles > 13 mm in diameter (12.8 ± 2.8 vs 8.3 ± 3.5; *p* < 0.01) and higher peak E2 levels (15,161 ± 4975 pmol/L vs 9659 ± 4534 pmol/L; *p* < 0.02) on day of trigger, and significantly more oocytes (16.1 ± 3.3 vs 8.6 ± 4.3; *p* < 0.01) and mature oocytes (12.1 ± 2.2 vs 6.7 ± 3.5; *p* < 0.01) yields, as compared to women who achieved same or more oocytes in the 2^nd^ attempt. No in between group differences were observed in women age, the duration of OS, total dose of gonadotropin used, nor peak progesterone levels.

### Women with no change in the daily gonadotropin dose in the 2nd IVF cycle attempt

While comparing the OS characteristics during the 1st IVF cycle attempt, those who achieved a lower oocyte yield in the 2nd attempt had significantly higher number of follicles > 13 mm in diameter (11.6 ± 5.1 vs 7.4 ± 3.8; *p* < 0.01) and higher peak E2 levels (12,312 ± 8860 pmol/L vs 7357 ± 4241 pmol/L; *p* < 0.005) on day of trigger, and significantly more oocytes (19.1 ± 6.8 vs 7.9 ± 4.4; *p* < 0.001) and mature oocytes (14.3 ± 5.3 vs 6.4 ± 3.7; *p* < 0.001) yields, as compared to women who achieved same or more oocytes in the 2nd attempt. No in between group differences were observed in women age, the duration of OS, total dose of gonadotropin used, nor peak progesterone levels.

### Women with increased daily gonadotropin dose in the 2nd IVF cycle attempt

While comparing the OS characteristics during the 1st IVF cycle attempt, those who achieved a lower oocyte yield in the 2nd attempt were significantly younger (35.1 ± 2.3yrs vs 36.4 ± 2.2yrs; *p* < 0.001), had significantly higher number of follicles > 13 mm in diameter (9.8 ± 3.9 vs 7.3 ± 3.9; *p* < 0.001), higher peak E2 (9560 ± 5636 pmol/L vs 7221 ± 4505 pmol/L; *p* < 0.01) and progesterone (2.8 ± 1.4 nmol/L vs 2.3 ± 1.2 nmol/L; *p* < 0.04) levels on day of trigger, and significantly more oocytes (12.7 ± 6.5 vs 7.6 ± 4.8; *p* < 0.001) and mature oocytes (9.9 ± 4.5 vs 5.2 ± 2.7; *p* < 0.001) yields, as compared to women who achieved same or more oocytes in the 2nd attempt. No in between group differences were observed in the duration of OS, nor the total dose of gonadotropin used.

### Women with optimal-high response (10 and more oocytes), as compare to those with low-suboptimal response (9 or less oocytes) in the 1st IVF cycle attempt

Women with optimal-high response were significantly younger (35.3 ± 1.9yrs vs 36.1 ± 2.4yrs; *p* < 0.001), had significantly higher number of follicles > 13 mm in diameter (11.1 ± 3.7 vs 6.36 ± 3.2; *p* < 0.001), higher peak E2 (11,107 ± 5963 pmol/L vs 6528 ± 3958 pmol/L; *p* < 0.001) and progesterone (3.2 ± 1.7 nmol/L vs 2.4 ± 1.7 nmol/L; *p* < 0.001) levels on day of trigger, and significantly more oocytes (15.4 ± 5.5 vs 5.7 ± 2.3; *p* < 0.001) and mature oocytes (11.4 ± 4.1 vs 4.6 ± 2.0; *p* < 0.001) yields, as compared to women with low-suboptimal response during the 1^st^ IVF cycle attempt. Moreover, while the total dose of gonadotropin used was significantly lower in the optimal-high responders (3032 ± 611 IU vs 3242 ± 814 IU, *p* < 0.04), no in between group difference was observed in the duration of OS. Moreover, while in the 2^nd^ IVF cycle attempt, the optimal-high responders received significantly lower daily gonadotropin dose (375 ± 90 IU vs 404 ± 109 IU, *p* < 0.04), compared to the low-suboptimal responders, no in between group differences were observed in the 2nd cycle attempt, in the duration of OS, peak E2 and progesterone levels on day of trigger, nor the number of oocytes (11.5 ± 6.9 vs 11.4 ± 7.2; *p* = NS) and mature oocytes (9.0 ± 4.8 vs 8.9 ± 5.6; *p* = NS).

Moreover, optimal-high responders that achieved a higher oocyte yield in the 2^nd^ attempt, had lower oocyte (13.4 ± 3.6 vs 16.3 ± 5.9, *p* < 0.01) and MII oocyte (9.5 ± 2.8 vs 12.3 ± 4.3, *p* < 0.001) yield in the 1^st^ cycle attempt, compared to those with same or lower yield in the 2^nd^ attempt. No in between group differences were observed in patients age, duration of OS, total dose of gonadotropin used, nor peak estradiol progesterone levels.

When exploring the performance of the low-suboptimal responders, those that achieved a higher oocyte yield in the 2nd attempt were older (36.5 ± 2.1yrs vs 35.2 ± 2.7yrs, *p* < 0.01), had lower oocyte (5.2 ± 2.3 vs 6.6 ± 2.1, *p* < 0.01) and MII oocyte (4.1 ± 1.8 vs 5.7 ± 2.0, *p* < 0.001) yield in the 1st cycle attempt, compared to those with same or lower yield in the 2nd attempt. No in between group differences were observed in the duration of OS, total dose of gonadotropin used, nor peak estradiol progesterone levels.

### Women with high peak E2 levels (> 9175 pmol/L), as compare to those with peak E2 levels < 9175 pmol/L.

Women with high peak E2 had significantly higher number of follicles > 13 mm in diameter (11.5 ± 3.7 vs 7.1 ± 3.6; *p* < 0.001), higher peak E2 (14,481 ± 5004 pmol/L vs 5520 ± 2127 pmol/L; *p* < 0.001) and progesterone (3.6 + 1.8 nmol/L vs 2.3 ± 1.5 nmol/L; *p* < 0.001) levels on day of trigger, and significantly more oocytes (13.8 ± 5.8 vs 8.6 ± 6.0; *p* < 0.001) and mature oocytes (10.5 ± 4.6 vs 6.5 ± 4.1; *p* < 0.001) yields, as compared to women with peak E2 level < 9175 pmol/L during the 1st IVF cycle attempt. Moreover, the proportion of patients with same or more oocytes in the 2nd IVF cycle attempt was significantly lower in patients with high peak E2 levels (38.3% vs 66.2%, *p* < 0.001), compared to those with peak E2 levels < 9175 pmol/L. In both groups, 27% of patients received the same daily gonadotropin dose in the second IVF cycle attempt.

When assessing only the subgroup of patients with high peak E2 (> 9175 pmol/L), those that achieved a higher oocyte yield in the 2nd attempt had significantly higher basal Day-3 FSH (8.4 ± 2.4 IU/L vs 7.1 ± 1.9 IU/L, *p* < 0.02) and FSH/LH ratio (1.8 ± 0.8 vs 1.5 ± 0.5, *p* < 0.03) and lower oocyte (10.7 ± 4.7 vs 15.8 ± 5.5, *p* <  > 0.001) and mature oocytes (7.9 ± 3.7 vs 12.1 ± 4.3, *p* < 0.001) yields. Moreover, with a cut-off of 9 oocytes, 78.8% of those with > 9 oocytes and 61.8% of those with < 9 oocytes will achieve lower/higher oocytes yield in the 2nd IVF cycle attempt, respectively.

## Discussion

In the present study of women undergoing 2 successive IVF cycle attempts for EEF, increasing the daily gonadotropin dose above 300 IU results in higher mature oocytes yield. This observation is in accordance with Drakopoulos et al. [14] who evaluated the ovarian response of suboptimal responders in terms of the number of oocytes retrieved, following their second IVF cycle. They evaluated exclusively patients who had the same stimulation protocol and used the same or higher initial dose of the same type of gonadotropin compared to their previous failed IVF attempt. According to their analysis, a dose increment of FSH remained the only significant predictor of the number of oocytes retrieved in the subsequent IVF cycle, with an increase of 50 IU of the initial rFSH dose leading to 1 more oocyte.

In the present study, a reduced oocyte yield in the 2^nd^ cycle attempt was observed in those who highly responded in the 1^st^ attempt, regardless the daily dose in the 2^nd^ cycle attempt (whether it was increased, no change and decreased). Moreover, the proportion of patients with same or more oocytes in the 2nd IVF cycle attempt was significantly lower in patients with high peak E2 levels, compared to those with peak E2 levels < 9175 pmol/L. In both groups, 27% of patients received the same daily gonadotropin dose in the second IVF cycle attempt. Furthermore, the lower oocyte yield in the 2nd attempt was received despite receiving significantly higher daily gonadotropins dose.

Among patients with high peak E2 (> 9175 pmol/L), those who achieved a lower oocytes yield in the 2^nd^ IVF cycle attempt had lower basal Day-3 FSH/LH ratio (1.5 ± 0.5 vs 1.8 ± 0.8, *p* < 0.03) and higher oocyte (range: 7–28, median:10; vs range: 2–15, median:7) and mature oocytes yields. With a cut-off of 9 oocytes, 78.8% of those with > 9 oocytes and 61.8% of those with < 9 oocytes will achieve lower/higher oocytes yield in the 2^nd^ IVF cycle attempt, respectively.

A major strength of our study is that we compared the IVF outcome in same cohort of "healthy" patients, undergoing two successive IVF cycle attempts for elective egg freezing (EEF). The fact that all women that participated in our study had two consecutive treatment cycles helps to eliminate multiple bias factors and to attribute the study results daily gonadotropin dose. On the other hand, our observation that in the 2nd cycle attempt, a significant percentage of women with good responses in 1st cycles, achieved lower oocyte numbers, even if dosage of gonadotropins was the same may be biases, either because of statistical return to the mean, differences in time periods between the two cycles or other potential biases inherent in a retrospective analysis of IVF cycles.

To conclude, patients attempting social/elective egg freezing has spread globally. Ovarian stimulation with high daily gonatotropin doses (300 IU) should be offered to this group of patients in their 1^st^ IVF cycle attempt, aiming to achieve the maximal oocytes cohort with minimum IVF cycle attempts. Moreover, in their 2nd IVF cycle attempt, those with high peak E2 (> 9175 pmol/L) in the 1st attempt, and basal Day-3 FSH/LH ratio < 1.5 and/or more than 9 oocytes retrieved, should receive same OS protocol with no change in the daily gonadotropin dose, aiming to obtain the best results.

## Data Availability

The datasets used and/or analysed during the current study are available from the corresponding author on reasonable request.

## References

[1] Penzias AS (2004). Improving results with assisted reproductive technologies: individualized patient-tailored strategies for ovulation induction. Reprod Biomed Online.

[2] Bosch E, Broer S, Griesinger G, Grynberg M, Humaidan P, Kolibianakis E, Kunicki M, La Marca A, Lainas G, Le Clef N, Massin N, Mastenbroek S, Polyzos N, Sunkara S, Timeva T, Töyli M, Urbancsek J, Vermeulen N, Broekmans F, Ovarian Stimulation TEGGO (2020). ESHRE guideline: ovarian stimulation for IVF/ICSI. Hum Reprod Open.

[3] Lensen SF, Wilkinson J, Leijdekkers JA, La Marca A, Mol BWJ, Marjoribanks J, Torrance H, Broekmans FJ. Individualised gonadotropin dose selection using markers of ovarian reserve for women undergoing in vitro fertilisation plus intracytoplasmic sperm injection (IVF/ICSI). Cochrane Database Syst Rev. 2018;2(2):CD012693. 10.1002/14651858.CD012693.pub2. 10.1002/14651858.CD012693.pub2 PMC6491064 29388198

[4] Klinkert ER, Broekmans FJ, Looman CW, Habbema JD, te Velde ER (2005). Expected poor responders on the basis of an antral follicle count do not benefit from a higher starting dose of gonadotrophins in IVF treatment: a randomized controlled trial. Hum Reprod.

[5] Manzi DL, Thornton KL, Scott LB, Nulsen JC (1994). The value of increasing the dose of human menopausal gonadotropins in women who initially demonstrate a poor response. Fertil Steril.

[6] Stadtmauer L, Ditkoff EC, Session D, Kelly A (1994). High dosages of gonadotropins are associated with poor pregnancy outcomes after in vitro fertilization-embryo transfer. Fertil Steril.

[7] Karande VC, Jones GS, Veeck LL, Muasher SJ (1990). High-dose follicle-stimulating hormone stimulation at the onset of the menstrual cycle does not improve the in vitro fertilization outcome in low-responder patients. Fertil Steril.

[8] Hofmann GE, Toner JP, Muasher SJ, Jones GS (1989). High dose FSH ovarian stimulation in low responder patients for in vitro fertilization. J In Vitro Fert Embryo Transf.

[9] Land JA, Yarmolinskaya MI, Dumoulin JC, Evers JL (1996). High-dose human menopausal gonadotropin stimulation in poor responders does not improve in vitro fertilization outcome. Fertil Steril.

[10] Haas J,  Zilberberg E, Kedem A, Dar S,  Orvieto R (2015). Do poor-responder patients benefit from increasing the daily gonadotropin dose from 300 to 450 IU during controlled ovarian hyperstimulation for IVF?. Harefuah.

[11] Ben-Rafael Z, Strauss JF, Mastroianni L, Flickinger GL (1986). Differences in ovarian stimulation in human menopausal gonadotropin treated wonien may be related to FSH accumulation. Fertil Steril.

[12] Benadiva CA, Ben-Rafael Z, Strauss JF, Mastroianni L, Flickinger GL (1988). Ovarian response of individuals to different doses of human menopausal gonadotropin. Fertil Steril.

[13] Ethics Committee of the American Society for Reproductive Medicine (2018). Planned oocyte cryopreservation for women seeking to preserve future reproductive potential: an Ethics Committee opinion. Fertil. Steril.

[14] Drakopoulos P, Santos-Ribeiro S, Bosch E, Garcia-Velasco J, Blockeel C, Romito A, Tournaye H, Polyzos NP (2018). The Effect of Dose Adjustments in a Subsequent Cycle of Women With Suboptimal Response Following Conventional Ovarian Stimulation. Front Endocrinol.

